# Reduced Cortical Surface Area in the Frontal Operculum as a Causal Risk Predictor for Chronic Pain

**DOI:** 10.1155/prm/4687197

**Published:** 2025-06-05

**Authors:** Xiuzhi Wang, Yipeng Le, Xichen Wang, Yingchao Song, Qian Su, Xiaoxiao Xiao, Yifan Li, Wen Qin, Chunshui Yu, Meng Liang

**Affiliations:** ^1^School of Medical Technology, School of Medical Imaging, Tianjin Key Laboratory of Functional Imaging, The Province and Ministry Cosponsored Collaborative Innovation Center for Medical Epigenetics, Tianjin Medical University, Tianjin 300070, China; ^2^College of Artificial Intelligence and Big Data for Medical Sciences, Shandong First Medical University & Shandong Academy of Medical Sciences, Shandong 250117, China; ^3^Department of Molecular Imaging and Nuclear Medicine, Tianjin Medical University Cancer Institute and Hospital, National Clinical Research Center for Cancer, Tianjin Key Laboratory of Cancer Prevention and Therapy, Tianjin's Clinical Research Center for China, Tianjin 300060, China; ^4^Department of Radiology, State Key Laboratory of Experimental Hematology, Tianjin Key Lab of Functional Imaging & Tianjin Institute of Radiology, Tianjin Medical University General Hospital, Tianjin 300052, China

## Abstract

Chronic pain is a prevalent and debilitating condition that imposes substantial personal and societal burdens. Despite its significance, the neural mechanisms underlying individual susceptibility to chronic pain remain inadequately understood. In this study, we examined the prospective associations between 1325 brain structural imaging phenotypes and the future risk of developing chronic pain in a UK Biobank cohort of 5754–5756 participants. These phenotypes encompassed regional and tissue volume, cortical surface area and thickness. General linear models (GLMs) were employed to identify brain structural variations associated with the risk of developing chronic pain, and then Mendelian randomization (MR) was employed to explore potential causal relationships between brain structure and chronic pain development. GLMs identified three significant associations between imaging phenotypes and the future development of chronic pain. All three imaging phenotypes pertained to the cortical surface area of the frontal operculum, albeit derived from three different brain atlases. Specifically, reduced cortical surface area in the frontal operculum was significantly associated with an increased risk of developing chronic pain: BA atlas area 44 (*T*=−4.10, *p*=4.24 × 10^−5^), Desikan atlas pars opercularis (*T*=−4.21, *p*=2.55 × 10^−5^), and DKT atlas pars opercularis (*T*=−3.96, *p*=7.47 × 10^−5^). Subsequent MR analysis further demonstrated a causally protective effect of larger cortical area in the prefrontal operculum against the risk of developing chronic pain (OR = 0.91, *p*=1.91 × 10^−2^). These results indicate a critical role of the surface area of frontal operculum in individual chronic pain susceptibility and provide a potential risk predictor for chronic pain development.

## 1. Introduction

Chronic pain is a prevalent and debilitating condition, affecting over 30% of the global population and imposing substantial personal, social, and economic burdens [1–3]. Despite its significant impact, the mechanisms underlying individual susceptibility to chronic pain remain poorly understood, hindering the development of effective prevention and treatment strategies.

Growing evidence suggests a role of brain structural alterations in the pathophysiology of chronic pain. A recent large-scale cross-sectional study identified significant associations between gray matter volume (GMV) atrophy in the hippocampus and subcallosal cortex and multisite chronic pain and a mediation effect of hippocampal GMV on the relationship between chronic pain and fluid intelligence [4]. Building on this, the same research group further revealed that hippocampal atrophy in patients with chronic musculoskeletal pain was correlated with accelerated brain aging [5]. Beyond cross-sectional evidence, the findings from longitudinal studies further emphasized the importance of brain structural changes in the development and progression of chronic pain. In a three-year follow-up study, Apkarian et al. tracked 39 individuals with acute pain, of whom 23 developed chronic pain while the rest recovered. Comparisons between the two groups demonstrated that smaller amygdala volume is an independent risk factor for chronic pain development [6]. Similarly, a recent longitudinal cohort study reported that reduced nucleus accumbens volume was linked to a higher risk of chronic pain development [7]. Together, these findings suggest that volume reductions in subcortical regions may underlie the increased susceptibility to chronic pain.

However, these previous studies on the relationship between brain risk factors and chronic pain development have been limited by small sample sizes and a narrow range of brain imaging phenotypes, resulting in a lack of a comprehensive understanding of the brain mechanisms underlying chronic pain. Moreover, while these findings have provided valuable insights, most studies were correlational studies, making it difficult to establish a causal relationship between brain structural alterations and chronic pain susceptibility.

To address these gaps, we leveraged the extensive neuroimaging and phenotypic data from the UK Biobank (UKBB), a large-scale population-based cohort (*n*> 5000), to systematically investigate the associations between 1325 baseline brain structural imaging-derived phenotypes (IDPs) and the future development of chronic pain. In addition, we applied Mendelian randomization (MR) analysis to make causal inferences of the identified significant associations between brain IDPs and chronic pain development.

## 2. Method

**Figure 1:**
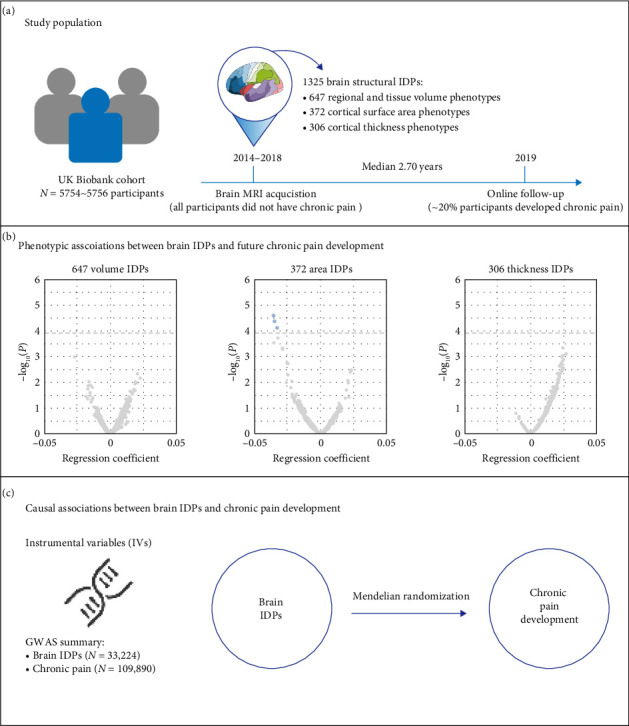
Study design and analytical framework for investigating the associations of brain structure with chronic pain development. (a) The study population. All participants used in the present study were from the UK Biobank cohort (*N* = 5754–5756). The brain MRI data were acquired between 2014 and 2018, resulting in 1325 brain imaging-derived phenotypes (IDPs) including 647 volume, 372 cortical surface area, and 306 cortical thickness phenotypes. Online follow-up questionnaires were collected in 2019 (the median interval between image acquisition and online follow-up was 2.70 years). All included participants did not have chronic pain at the time of brain MRI data acquisition, and around 20% of them developed chronic pain at the time of online follow-up. (b) Phenotypic associations between 1325 baseline brain IDPs and future chronic pain development. (c): Mendelian randomization for making causal inference between baseline brain IDPs and future chronic pain development. The instrumental variables (IVs) were derived from a GWAS summary statistics for brain IDPs (*N* = 33,224) and a GWAS for chronic pain development (*N* = 109,890).

The study design, participant details, and analytical framework are illustrated in Figure 1.

### 2.1. UKBB Cohort

The UKBB is a large, prospective cohort study designed to collect extensive genetic and phenotypic data from participants across the United Kingdom. Initiated in 2006, the study recruited approximately 500,000 middle-aged individuals (aged 40–69 years) from 22 assessment centers located in Scotland, England, and Wales. Following the first data collection, subsets of participants were invited for follow-up assessments, which included imaging data collection and the completion of online questionnaires. The data utilized in this study are described based on their collection time points as follows:

1. First data collection (2006–2010, *n* = 502,415): during the initial phase, 502,415 participants were recruited. Data were collected using touchscreen questionnaires, oral interviews, body measurements, and blood samples. In this study, DNA extracted from blood samples were used in the GWAS for chronic pain.
2. Image data collection (2014–2020, *n* = 42,801): a subset of 42,801 participants who had completed the first data collection underwent structural and functional brain MRI scans between 4 and 14 years after the first data collection (mean interval: 9 years). In this study, 1325 MRI-derived brain structural phenotypes were analyzed.
3. Online follow-up (2019–2020, *n* = 165,876): a total of 165,876 participants completed the online questionnaire, “Experience of Pain,” approximately 8–14 years after first data collection (mean interval: 10 years). The questionnaire comprised 10 subsections designed to capture information on the presence and duration of chronic pain, its location, nature, and intensity over the last 24 h.

### 2.2. Chronic Pain Definition

The definition of chronic pain status at the time of brain MRI data acquisition was based on UKBB field ID 6159, which recorded participants' answers to the following questions: (1) “In the last month, have you experienced any of the following that interfered with your usual activities? (You can select more than one answer).” The participants could choose from the following 9 options: “Headache,” “Facial pain,” “Neck or shoulder pain,” “Back pain,” “Stomach or abdominal pain,” “Hip pain,” “Knee pain,” “Pain all over the body” and “None of the above.” Participants who answered “None of the above” were categorized as having “no pain.” (2) For those reporting pain at one of the eight listed body sites pain, a follow-up question was asked: “Have you had this body site pain for more than 3 months?” Participants answering “Yes” were classified as having “chronic pain” while those responding “No” were categorized as having “acute pain.” To maximize the sample size, participants with “no pain” or “acute pain” at the time of brain MRI data acquisition were included in the present study, whereas those with “chronic pain” were discarded.

Chronic pain status at the online follow-up was determined using the UKBB's “Experience of Pain” questionnaire (https://biobank.ndph.ox.ac.uk/showcase/ukb/docs/pain_questionnaire.pdf). During online follow-up, participants were asked: “Are you troubled by pain or discomfort, either all the time or on and off, that has been present for more than 3 months?” Those who responded “yes” were classified as having chronic pain and were further asked to specify the duration, choosing from the following options: “3–12 months,” “1–5 years,” “more than 5 years,” “do not know,” or “prefer not to answer.”

### 2.3. Participants Selection

**Figure 2:**
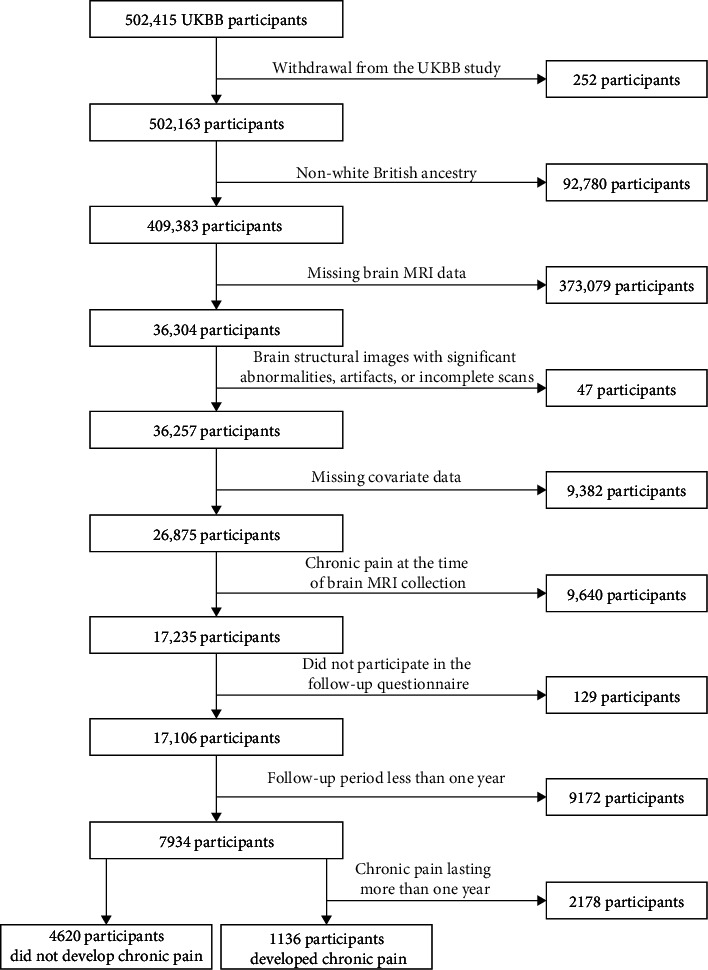
Participant selection flowchart.

To determine the final participants included in the subsequent association analyses and MR analyses, the following exclusion criteria were applied: (1) withdrawal from the UKBB study; (2) non-White British ancestry; (3) missing brain MRI data; (4) significant abnormalities, artifacts, or incomplete scans in brain MRI images; (5) missing covariate data; (6) chronic pain at the time of brain MRI collection; (7) missing follow-up questionnaire data collection; (8) the interval between MRI data acquisition and online questionnaire data collection less than 1 year; and (9) chronic pain having lasted more than 1 year at the time of online questionnaire data collection. The combination of criteria 8 and 9 ensured that the development of chronic pain occurred after brain MRI acquisition for all included participants with chronic pain, minimizing the risk of misclassifying chronic pain as acute pain at baseline (i.e., MRI data acquisition stage). According to the abovementioned exclusion criteria, the participants who remained free of chronic pain at the online follow-up were also included. After the quality control process of participants, brain MRI data and chronic pain measures, a total of 5756 participants were included in the association analyses between 1325 brain structural IDPs and chronic pain development (the detailed participants information is shown in Figure 2). All included participants were free of chronic pain at the acquisition of brain MRI data, and approximately 20% of these participants developed chronic pain during the online follow-up (Figure 2).

### 2.4. MRI Data Acquisition, IDP Extraction, and Quality Control

In this study, we utilized 1325 brain structural IDPs, including 647 regional and tissue volume phenotypes, 372 cortical surface area phenotypes, and 306 cortical thickness phenotypes. Detailed description for 1325 IDPs can be found in Supporting Table 1. These IDPs were generated from MRI data acquired at four scanning centers: Cheadle, Reading, Newcastle, and Bristol. All scans were conducted using Siemens Skyra 3T MRI scanners with identical configurations and 32-channel radiofrequency head coils. Comprehensive details about the scanning protocols and procedures are provided in the UKBB documentation (https://biobank.ndph.ox.ac.uk/showcase/showcase/docs/brain_mri.pdf).

Brain IDPs' quality control were performed as follows: first, IDP values exceeding six times the median absolute deviation from the median were considered as outliers and excluded; then, to account for scanner-related variability, we applied ComBat harmonization [8], followed by normal score transformation to standardize IDP values and ensure a Gaussian distribution [9]. Notably, this quality control process was conducted independently for each IDP to maximize the sample size available for the association analysis of each IDP. After quality control, the final sample sizes for all IDPs were 5756, with a few exceptions of 5754 (the exact sample size for each IDP is provided in Supporting Table 1).

### 2.5. Association Analyses Between Baseline Values of 1325 IDPs and Future Chronic Pain Development

General linear models (GLMs) implemented in R (Version 4.3.1) [10] was used to identify associations between baseline values of 1325 IDPs and future development of chronic pain. The interval between the imaging data acquisition and chronic pain questionnaire data acquisition had a median value of 2.70 years (ranging from 1.00 to 5.02 years). All continuous variables were standardized to a mean of 0 and a variance of 1 before GLM analyses. Given the substantial correlation among the 1325 IDPs, we calculated an effective number of IDPs for multiple comparisons correction, using an established method [11, 12]. Specifically, we generated a correlation matrix of the 1325 IDPs, computed its eigenvalues, and derived the effective number of independent phenotypes (*M*_eff_) from the eigenvalues: *M*_eff_=*M* − ∑_*i*=1_^*M*^[*I*(λ_*i*_ > 1)(λ_*i*_ − 1)], where *M* represents the total number of phenotypes (1325 in this study), λ_*i*_ is the *i*-th eigenvalue of the correlation matrix, *I*(*x*) is the indicator function that equals 1 if the condition (*x*) is true and 0 otherwise, and ∑_*i*=1_^*M*^[*I*(λ_*i*_ > 1)(λ_*i*_ − 1)] estimates the redundant number of tests by accounting for eigenvalues greater than 1. The calculated M_eff_ for the 1325 brain IDPs was 403.85, leading to a corrected threshold of *p* < 0.05/403.85=1.24 × 10^−4^.

Each IDP was assessed in a separate GLM to evaluate its association with future chronic pain development (IDP was the independent variable and chronic pain development was the dependent variable). All association analyses were adjusted for age (at brain MRI data acquisition), sex, age^2^, age × sex, age^2^× sex, and the first 10 genetic principal components (PCs), total intracranial volume (TIV), follow-up interval, and seven other covariates which have been suggested to be related to chronic pain, including BMI, multiple deprivation index, smoking status, alcohol consumption status, educational attainment, analgesic usage, and history of seeking medical advice for nervousness, anxiety, tension, or depression.

### 2.6. GWAS for Chronic Pain Development

To perform MR for the causal inference between brain IDPs and chronic pain development, GWAS summary statistics of the brain IDPs and chronic pain development are required. Due to the absence of chronic pain GWAS summary statistics to date, we performed a case/control GWAS of chronic pain development using the following procedure.

The detailed processing and imputation procedures for the genetic data of the UKBB were outlined in a previous study [13]. In the GWAS for chronic pain development in the present study, samples were removed using the following criteria: (1) having brain MRI data to ensure there was no sample overlap between the GWAS for brain IDPs and the GWAS for chronic pain development; (2) withdrawn from UKBB project; (3) non-White British ancestry (field ID: 22,006, sample selected based on self-reported ancestry and genetic PC); (4) sex chromosome aneuploidy; (5) sex mismatch; (6) outliers for heterozygosity or missing rate; and (7) kinship coefficient > 0.0884 [14]. Variants were removed using the following criteria: (1) minor allele frequency < 0.001; (2) Hardy–Weinberg equilibrium *p* < 1 × 10^−7^; (3) imputation quality score < 0.3; and (4) rsid duplicated. After applying these quality control measures, a total of 109,890 participants (mean age = 66.8, SD = 7.7) and 13,833,322 variants remained in the subsequent GWAS analyses.

Logistic regression model of PLINK (v.2.0) [15] was used to perform GWAS of chronic pain development. The model included age, sex, age^2^, age × sex, age^2^× sex, and the first 40 PCs as covariates. Prior to conducting the logistic regression analysis, quantitative covariates were standardized to have a mean of 0 and a variance of 1.

### 2.7. MR

Three brain IDPs were identified to be significantly associated with future chronic pain development in the above GLM analyses. The TwoSampleMR package (Version 0.6.8) [16] in R was then employed to formally test the causal effects of the 3 predictive brain IDPs on chronic pain development. The GWAS summary data for chronic pain development was derived from the GWAS described above in the present study (*n*= 109,890). The GWAS summary statistics for the 3 brain IDPs (the cortical surface areas of BA-exvivo rh area BA44; aparc-Desikan rh area parsopercularis; aparc-DKTatlas rh area parsopercularis) were previously published and obtained from the UK Biobank cohort (*n*= 33,224) [17]. Instrumental variants (IVs) were initially defined as the SNPs associated with the exposure (brain IDP) at *p* < 1 × 10^−5^, a threshold commonly used in previous MR studies to ensure a sufficient number of IVs and adequate association strength for each exposure [18–21]. Independent IVs were then identified through LD clumping with a window size of 10,000 kb and an LD threshold of *r*^2^ < 0.001. Subsequently, the effect alleles of the IVs were harmonized between the exposure and outcome datasets, with palindromic IVs excluded to avoid strand ambiguity. The F-statistics for all IVs were calculated, and IVs with F-statistic values less than 10 were removed to minimize the risk of weak instrument bias [22]. In addition, Steiger's test [23] was performed to exclude IVs that exhibited a stronger correlation with the outcome than with the exposure. Finally, outliers were identified and excluded using the RadialMR method [24].

Five MR approaches, including inverse variance weighting (IVW), maximum likelihood, MR-Egger, weighted median, and weighted mode, were used to investigate the causal effect of brain IDPs on chronic pain development. The IVW method was chosen as the primary approach for MR analyses, as it provides the most efficient causal estimates under the assumption that all IVs are valid, offering the highest statistical power [25]. A causal effect was considered significant if the IVW estimate had a *p* < 0.05/1.18 (Meff for 3 predictive brain IDPs) = 4.2 × 10^−2^ and if its direction was consistent with the results from the other 4 MR methods. To ensure the reliability of the findings, Cochran's *Q* test was used to investigate heterogeneity in the MR estimates across IVs, and MR-Egger intercepts were assessed to evaluate bias due to weak IVs and the potential for horizontal pleiotropy.

## 3. Results

### 3.1. Associations Between 1325 Baseline Brain IDPs and Future Chronic Pain Development

**Figure 3:**
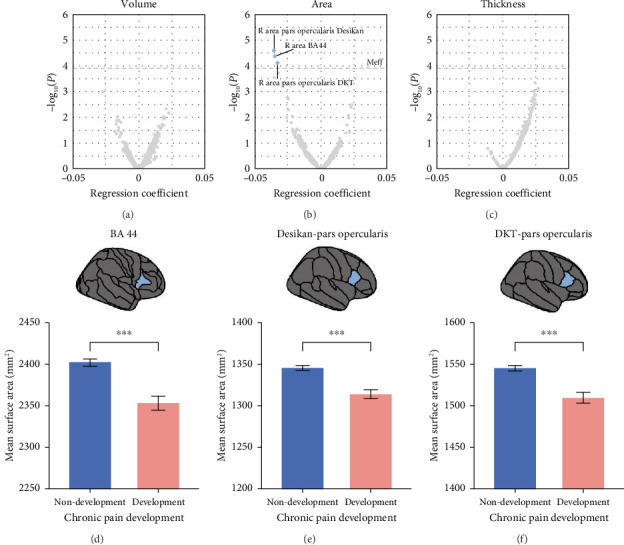
The results of the association analyses between 1325 baseline brain structural IDPs and future chronic pain development. Panels (a–c) depict the correlation analyses for brain regional volume (a), cortical surface area (b), and cortical thickness (c) phenotypes with future chronic pain development. The *x*-axes show the regression coefficients, and the *y*-axes represent the −log_10_(P) values. The horizontal dashed lines mark the threshold for statistical significance (*p*< 0.05/403.85 = 1.24 × 10^−4^). Blue dots indicate statistically significant correlations whereas gray dots represent nonsignificant correlations. Panels (d–f) show the significant differences in the cortical surface area of the frontal operculum region defined using three brain atlases (panel (d) BA atlas; panel (e) Desikan atlas; and panel (f) DKT atlas) between participants without and with chronic pain. Error bars represent the standard error of the mean (SEM). Statistical significance is indicated as follows: ^∗^*p* < 0.05, ^∗∗^*p* < 0.01, and ^∗∗∗^*p* < 0.001. Detailed results are provided in Supporting Table 1. Abbreviation: L, left; R, right.

GLM analyses identified three brain IDPs showing significant negative associations with chronic pain development (*p* < 1.24 × 10^−4^). All three IDPs involved the cortical surface area of the right frontal operculum cortex, segmented using three different atlases (pars opercularis from the Desikan atlas, *T*=-4.21, *p*=2.55 × 10^−5^; area 44 from the BA atlas, *T*=−4.10, *p*=4.24 × 10^−5^; and pars opercularis from the DKT atlas, *T*=−3.96, *p*=7.47 × 10^−5^) (Figure 3). These three negative associations convergently indicate that the reduction of surface area of the right frontal operculum cortex was linked with a higher risk of developing chronic pain in the future. Detailed results of all 1325 IDPs were shown in Supporting Table 1.

### 3.2. MR

**Figure 4:**
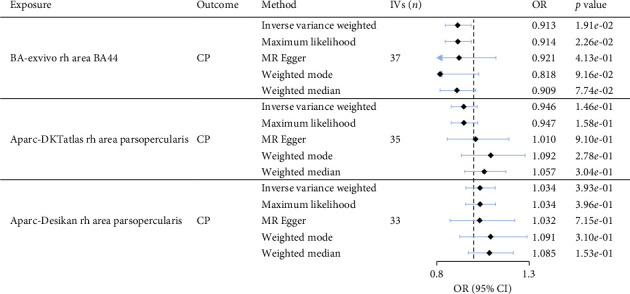
The results of MR analyses for testing causal effects of the cortical surface area of the frontal operculum defined using three brain atlases on chronic pain. Five approaches (inverse variance weighted, maximum likelihood, MR Egger, weighted mode, and weighted median) were used with the inverse variance weighted method as the primary method. The black dots represent the odds ratios (ORs), the blue horizontal lines indicate the 95% confidence intervals (CIs), and the arrows denote instances where the confidence intervals extend beyond the axis range. *p*< 4.23 × 10^−2^. Abbreviation: CP, chronic pain; IV, instrumental variables.

The GWAS for chronic pain development identified two genome-wide significant SNPs, which were consolidated into one distinct genomic locus represented by a single lead SNP (Supporting Table 2. *p* < 5 × 10^−8^). The results of the MR analyses are shown in Figure 4. Among the three significant associations identified by GLM analyses, the cortical surface area of the right frontal operculum defined using the BA atlas was found to have a significant causal effect on chronic pain development using the IVW method (OR = 0.913, *p*=1.90 × 10^−2^) and the sign of this effect was consistent with that obtained from the other 4 MR approaches. In addition, the MR-Egger intercept test showed no evidence of directional pleiotropy in the significant causal effect (Egger intercept = 0.00047, *p*=0.93), suggesting that the IVs used in MR analysis were not significantly influenced by horizontal pleiotropy. In addition, Cochran's *Q* test for MR-IVW showed no significant heterogeneity among the IVs (*Q*=28.4, df=35, and *p*=0.78), indicating consistency in the effects across the IVs. These results suggest that the decreased area of the right frontal operculum was causally related to an increased risk for the development of chronic pain.

## 4. Discussion

This study provides a comprehensive investigation into the prospective and causal associations between baseline brain structural imaging phenotypes and future chronic pain development. The strengths of our study are three folds: first, compared with previous studies, we leveraged a large dataset consisting of over 5000 participants in the UKBB and analyzed 1325 brain structural IDPs (including cortical volume, thickness, and surface area) and thus were able to offer a more robust and more complete picture of the relationship between brain structure and chronic pain development. Second, the use of a prospective cohort with the collection of MRI data prior to the development of chronic pain ensured a clear temporal order between brain structure measurement and chronic pain development and thus laid a more solid foundation for testing the causal relationship between them. Third, by combining prospective association analysis with MR, we were able to test the causal effect of brain structure on the development of chronic pain. Building on these strengths, we identified reduced cortical surface area in the right frontal operculum as a significant predictor of chronic pain development, and the causal relationship between them was further verified using MR analyses. These findings underscore the critical role of the cortical surface area of the right frontal operculum in affecting an individual's susceptibility to developing chronic pain. Importantly, the identification of the right frontal operculum as a risk predictor for chronic pain susceptibility lays the foundation for developing new strategies for early screening of individuals with high risk of developing chronic pain as well as for targeted interventions—such as transcranial magnetic stimulation or cognitive-behavioral therapy—to enhance beneficial neuroplastic changes, ultimately aiding in the prevention and management of chronic pain.

Among the 1325 prospective association analyses, we identified three significant correlations between baseline brain structure IDPs and the future development of chronic pain (Figure 3; Supporting Table 1). Notably, all three IDPs were linked to the cortical surface area of the frontal operculum, with a reduced surface area of this region associated with an increased risk of chronic pain. The frontal operculum is known to play a crucial role in various cognitive and emotional processes associated with pain [26–30], including somatosensory integration, attention modulation, and emotional regulation. A reduced cortical surface area in the frontal operculum may indicate impairments in these processes, leading to dysregulated pain perception and processing, thereby increasing the risk of developing chronic pain. Importantly, the frontal operculum is a key component of the descending pain modulatory pathway—a network that also involves subcortical structures such as the amygdala and thalamus [31]. Abnormalities in these regions have been consistently reported in chronic pain populations [32–37]. This pathway is responsible for exerting top–down control over pain [38, 39], and structural deficits in the frontal operculum could change its connectivity with these subcortical regions, thereby compromising the efficiency of pain inhibition [30]. As a result, individuals with reduced opercular surface area may be less capable of modulating pain effectively, which in turn increases their susceptibility to developing chronic pain. Previous studies have reported reductions in other structural IDPs, such as GMV and cortical thickness, and decreased task-induced activation within the operculum cortex in populations with chronic back pain, headache, or chronic visceral pain [27, 40, 41]. Along with these previous findings, the observed reduced cortical surface area of the frontal operculum in our present study suggests that the operculum may be a critical region affected by multiple subtypes of chronic pain. In our present study, the brain IDPs were acquired at the baseline before the development of chronic pain, and we did not find significant associations between the GMV or cortical thickness of the frontal operculum, indicating that the cortical surface area of this region is a more sensitive predictor for the development of chronic pain than GMV and cortical thickness.

The results of the MR analyses further verified the causal effect of the cortical area of the frontal operculum cortex (BA44) on the risk of developing chronic pain (Figure 4). Specifically, a decreased surface area in the frontal operculum cortex was found to causally increase the risk of developing chronic pain. The role of the structure of frontal operculum in chronic pain was also suggested in a recent MR study which investigated the causal relationship between large-scale brain structural IDPs and migraine and revealed significant causal effects of both macrostructural features (e.g., cortical volume and thickness) and microstructural features (e.g., intrinsic curvature index and intracellular volume fraction) in the medial prefrontal and frontal opercular cortex on the occurrence of migraine [20]. The sensitivity analyses of the present study demonstrated that the observed significant causal effect was robust and not substantially influenced by potential confounding factors such as horizontal pleiotropy or heterogeneity. This suggests that the identified causal relationship was likely to be genuine and not artifacts of bias or unaccounted genetic variation.

This study has several limitations. First, we only found evidence for the cortical surface area of the frontal operculum to be associated with the risk of developing chronic pain. It is likely that other structural characteristics of different brain regions are also linked to the development of chronic pain but remained undetected in our study. A possible reason is that we did not differentiate between subtypes of chronic pain (e.g., chronic pain in different body sites), which might have hindered the detection of those brain structural IDPs specific to the development of certain subtypes of chronic pain and even obscuring the interpretation of our current finding regarding whether it applied to all subtypes. Second, the study focused exclusively on individuals of White British ancestry and also the UKBB cohort might have a healthy volunteer bias, which may limit the generalizability of the results to other populations. Future replication studies in independent datasets and more diverse cohorts—particularly those with various genetic backgrounds and clinical characteristics—are needed to confirm the generalizability of our results. In addition, as this study examined only structural phenotypes, future research should not only extend to brain functional IDPs but also incorporate longitudinal imaging studies tracking changes in brain structure and function to provide a more comprehensive and dynamic picture of alterations of brain IDPs with respect to chronic pain development. Moreover, while potential effects of sex on brain IDPs was adjusted as a covariate in the present study, sex differences in chronic pain prevalence is well known and sex-specific mechanisms underlying chronic pain development should be investigated in the future. Similarly, environmental factors might have interactive effects with brain structural risk on chronic pain development which deserves further investigation in the future.

## 5. Conclusion

In the present study, we identified the cortical surface area of the right frontal operculum as a predictor for the development of chronic pain and also provided causal evidence between this brain IDP and the development of chronic pain. These findings advance our understanding of the neural substrates of chronic pain susceptibility and offer promising directions for developing targeted interventions.

## Data Availability

The UKBB data used in this study were obtained under approved application number 75556. Access to the UK Biobank dataset is open to bona fide researchers upon application (https://www.ukbiobank.ac.uk/).

## Supporting Information

Supporting Table 1 presents the results of the phenotypic correlation analysis between 1325 brain IDPs and future chronic pain development.

Supporting Table 2 provides the genome-wide significant lead SNPs associated with chronic pain development.
